# Tumor Growth Mitigating Effects of Valproic Acid in Systemic Malignancies

**DOI:** 10.1155/2015/540183

**Published:** 2015-07-28

**Authors:** Shailendra Kapoor

**Affiliations:** Private Practice, Richmond, VA 23114, USA

Recent data suggests that valproic acid (VPCA) also attenuates and limits tumor growth in a number of systemic malignancies [1].

For instance, VPCA has a suppressive effect on tumor growth in renal malignancies. Oertl et al. have recently shown that VPCA mediates this role in part by altering the expression of beta1 integrins in tumor cells [2]. VPCA also mediates this role in part by upregulating BAX expression. These changes have recently been confirmed in Caki-1 and KTC-26 cell lines. In addition, VPCA accentuates the expression of ULBP1 and ULBP2. Jones et al. have recently demonstrated that, at the same time, VPCA accentuates p21 expression [3]. Recent data also suggests that IFN-alpha accentuates the cytotoxic effects of VPCA. Cyclin B and cyclin D3 levels are also modulated by VPCA [4]. Yang et al. have recently demonstrated that VPCA also accentuates the cytotoxicity of NK cells against renal cancer cells [5]. In addition, VPCA also affects tumor cell adhesiveness thereby further attenuating tumor expansion in renal carcinomas [6].

Similarly, VPCA has an inhibitory effect on tumor growth in gastric malignancies. VPCA has a positive impact on acetyl-*α*-tubulin levels. It also mediates this role in part by attenuating c-Myc expression. Bcl-2 expression is downregulated concurrently. As a result, tumor cell apoptosis is markedly augmented. Zhao et al. have recently demonstrated that this is accompanied by accentuation of p21 (Waf/cip1) expression [7]. These changes have recently been confirmed in BGC-823, OCUM-2MD3, HGC-27, and SGC-7901 cell lines. In addition, VPCA has a negative impact on cyclin A as well as cyclin D1 expression. At the same time, tumor cell proliferation is markedly attenuated [8]. This is accompanied by accentuation of acetyl-histone H3 levels. G1 phase arrest is typically seen. In addition, Yagi et al. have recently demonstrated that survivin expression is significantly downregulated [9]. Mad1 expression is upregulated concurrently. p27 expression is also accentuated simultaneously. Besides the above-mentioned changes, VPCA also augments and enhances caspase 9 and caspase 3 activation thereby further abrogating tumor growth in gastric carcinomas.

As is obvious from the above discussion, VPCA exhibits potent tumor growth mitigating effects. Hopefully, the coming few years will see an increase in the utilization of VPCA as an antineoplastic agent.
